# Spatial patterns of bat diversity overlap with woodpecker abundance

**DOI:** 10.7717/peerj.9385

**Published:** 2020-06-18

**Authors:** Dorota Kotowska, Marcin Zegarek, Grzegorz Osojca, Andrzej Satory, Tomas Pärt, Michał Żmihorski

**Affiliations:** 1 Institute of Nature Conservation, Polish Academy of Sciences, Kraków, Poland; 2 Mammal Research Institute, Polish Academy of Sciences, Białowieża, Poland; 3 Department of Management and Logistics, Helena Chodkowska University of Technology and Economics, Warsaw, Poland; 4 Nature and Forest Research Office, Warsaw, Poland; 5 Department of Ecology, Swedish University of Agricultural Sciences, Uppsala, Sweden

**Keywords:** Bat communities, *Dendrocopos major*, Indicator species, Managed forest, Windthrow

## Abstract

Woodpecker diversity is usually higher in natural forests rich in dead wood and old trees than in managed ones, thus this group of birds is regarded as an indicator of forest biodiversity. Woodpeckers excavate cavities which can be subsequently used by several bird species. As a consequence, their abundance indicates high avian abundance and diversity in forests. However, woodpecker-made holes may be also important for other animals, for example, mammals but it has seldom been investigated so far. Here, we examine how well one species, the Great Spotted Woodpecker, predicts species richness, occurrence and acoustic activity of bats in Polish pine forests. In 2011 we conducted woodpecker and bat surveys at 63 point-count sites in forests that varied in terms of stand age, structure and amount of dead wood. From zero to five Great Spotted Woodpeckers at a point-count site were recorded. The total duration of the echolocation calls during a 10-min visit varied from 0 to 542 s and the number of bat species/species groups recorded during a visit ranged between zero to five. The local abundance of the woodpecker was positively correlated with bat species richness (on the verge of significance), bat occurrence and pooled bat activity. The occurrence of *Eptesicus* and *Vespertilio* bats and *Nyctalus* species was positively related with the abundance of the Great Spotted Woodpecker. The activity of *Pipistrellus pygmaeus, Eptesicus* and *Vespertilio* bats and a group of *Myotis* species was not associated with the woodpecker abundance, but echolocation calls of *Nyctalus* species, *P. nathusii* and *P.pipistrellus* were more often at sites with many Great Spotted Woodpeckers. Moreover, the probability of bat presence and the activity of bats was generally higher shortly after dusk and in middle of the summer than in late spring. We suggest that the observed correlations can be driven by similar roosting habitats (e.g., woodpeckers can provide breeding cavities for bats) or possibly by associated invertebrate food resources of woodpeckers and bats. The abundance of Great Spotted Woodpecker seems to be a good positive indicator of bat species richness, occurrence and activity, thus adding a group of relatively cryptic forest species that are indicated by the presence of the Great Spotted Woodpecker.

## Introduction

Woodpeckers (order: *Piciformes*, family *Picidae*) are birds of forests and woodlands that are largely dependent on the availability of old, dead and dying trees used for nesting, roosting and foraging. Among picids, there are some non-specialized species, which are less sensitive to the structural changes in forests (Angelstam & Mikusiński, 1994), however most of them are strictly linked with old-growth stands. Therefore, the highest abundance and diversity of woodpeckers is recorded in natural forests (like e.g., in the Białowieża Forest in eastern Poland, where all 10 European woodpecker species co-occur; Tomiałojć & Wesołowski, 2005).

Woodpeckers are also widely considered to be keystone species in forest ecosystems due to their nesting and foraging behavior related to cavity excavation. As primary cavity-nesters, woodpeckers excavate holes in trees which can be subsequently occupied by several non-excavator bird species, such as tits, nuthatches, starlings and flycatchers (Martin, Aitken & Wiebe, 2004). Most woodpeckers excavate one nesting hole each breeding season and, depending on the condition and species of the tree, a cavity can persist for more than 20 years (Wesołowski, 2011). As a consequence, we expect woodpecker abundance to be positively related to avian abundance and diversity in forests at both the stand and landscape levels (Drever et al., 2008; Mikusiński, Gromadzki & Chylarecki, 2001).

However, cavities in trees may also be of great importance for other animals, such as invertebrates (McComb & Noble, 1982; Nilsson & Baranowski, 1997), amphibians (Wells, 2007), reptiles (Webb & Shine, 1997) and mammals (e.g., dormice, squirrels and bats; Czeszczewik, Walankiewicz & Stańska, 2008; Jurczyszyn, 2007; Kalcounis & Brigham, 1998). Among these groups, bats are regarded as one of the most understudied vertebrate taxa occurring in forest habitats, despite the fact that great majority of European bat species use forests and forest edges for foraging and utilize trees as roosts for hibernation, maternity or as transitional shelters (Dietz & Kiefer, 2016). The forest bats generally prefer old stands with high availability of suitable shelters in large trunks, dead or dying trees (Kunz, Lumsden & Fenton, 2003; Lacki & Baker, 2003; Vonhof & Barclay, 1996). The roost types occupied by these species include natural cracks, gaps and crevices in the wood, as well as woodpecker cavities (Lučan, Hanák & Horáček, 2009; Regnery et al., 2013). Bats may also co-occur with birds in a single tree hole (Myczko et al., 2017). Nevertheless, very little is known about the extent to which bats are dependent upon the availability of woodpecker-made holes (Kunz, Lumsden & Fenton, 2003; Miller, Arnett & Lacki, 2003) and whether abundance of woodpeckers could be an indicator of high bat diversity.

In managed forests tree cavities can be limited for cavity-nesters and artificial nest-boxes often increase abundances of bats (Ciechanowski, 2005; Rachwald et al., 2018; Smith & Agnew, 2002). Thus, positive association between woodpeckers (cavity-providers) and bats (cavity-users) can be expected. Furthermore, resource heterogeneity resulting from abundance of old trees or availability of food (e.g., saproxylic insects) can cause abundance of woodpeckers to associate with abundance of bat species utilizing these or related resources, including those bat species not breeding in tree cavities. On the other hand, competition for available breeding or roosting sites may reduce any positive association between woodpeckers and bats (Sparks et al., 2003). However, to our knowledge, no studies on patterns of co-occurrence of woodpeckers and bats have been made so far.

Here we investigated the relationships between the abundance of the Great Spotted Woodpecker and indices of bat community in pine forests. The Great Spotted Woodpecker *Dendrocopos major* is the most common and widespread habitat generalist among European woodpeckers (Ćiković et al., 2014; Michalek & Miettinen, 2003). It inhabits a variety of forest types and excavates breeding holes in both dead and living trees. Thus, the presence of Great Spotted Woodpecker is especially important for secondary cavity-nesters in managed forests, where natural tree holes are scarce (Andersson et al., 2018). We investigated the links between the local abundance of the Great Spotted Woodpecker and local species richness, occurrence and flight activity of bats. We hypothesized that these associations should be positive, as both woodpecker and bats may be more abundant in forests of specific structure (e.g., Fontúrbel et al., 2015). Thus, woodpeckers may be negatively affected by human activity, such as cutting and removing old trees, but it seems they benefit from natural forest structure and its dynamics (Myczko et al., 2014), as well as from disturbances, like windthrows and fires (Żmihorski, 2010; Żmihorski et al., 2019). In our study, we therefore considered diverse forest habitat in terms of structure and amount of dead wood and, as a consequence, usefulness for woodpeckers. We expected that spatial patterns of bat diversity and activity will follow distribution of woodpecker, thus making the latter an useful indicator of this group of cryptic mammals.

## Materials and Methods

### Study area

**Figure 1:**
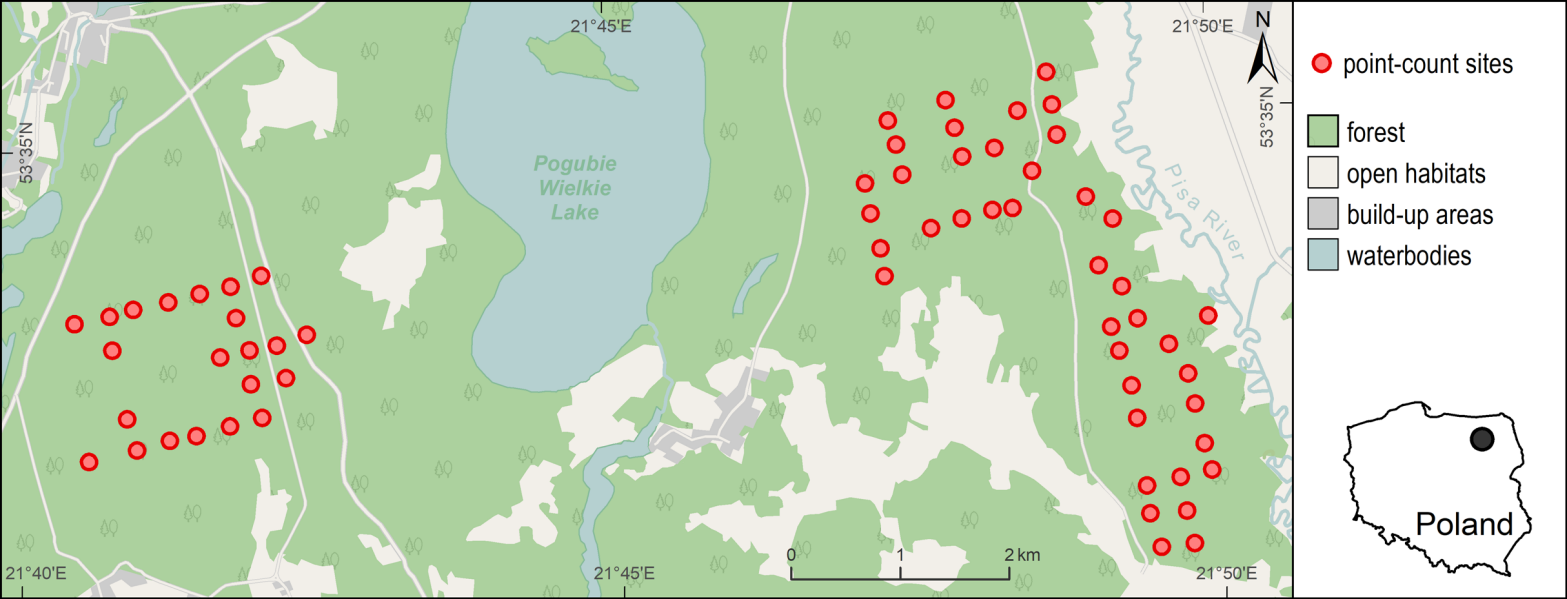
Distribution of 63 point-count sites in the Pisz Forest, NE Poland, sampled in 2011 for Great Spotted Woodpecker abundance, bat species richness, bat species occurrence and acoustic activity of bats. Background map data were provided by ©OpenStreetMap contributors.

The study was conducted in the Pisz Forest, located in a young glacial landscape of North-Eastern Poland (Fig. 1). This forest complex covers an area of ca. 90,000 ha. The terrain is generally flat (the altitude is between 110 and 180 m a.s.l.). The soils are mainly sandy and the tree stand is dominated by pine (*Pinus sylvestris*) with small amounts of spruce (*Picea abies*) and oak (*Quercus robur*). The age structure of the stand ranges from 0 to 150 years. The forest is state-owned and is managed for timber production by the Polish State Forests National Forest Holding. In July 2002 a strong wind severely damaged about 15,000 ha of the forest complex and created a mosaic of open areas, partially damaged stands with single trees or groups of trees and small areas of undisturbed forest. About 40% of the forest affected by the windstorm was completely destroyed and the total volume of damaged trees in the area was estimated at 2.5 million cubic meters (Dobrowolska, 2007). In the following years, large-scale salvage logging (fallen and broken trees removal) and artificial tree planting of the cleared areas was applied in the disturbed forest. However, a part of it (445 ha) was left unmanaged creating an experimental area of naturally regenerating windthrow.

The application of different management regimes resulted in highly variable forest structure at relatively small area (within just a few square kilometers), which created habitat patches potentially suitable for the Great Spotted Woodpecker (stands with dead wood and dying trees, old undisturbed stands) and unsuitable ones (e.g., salvaged windthrow, young pine plantations, etc., see Appendix S1). Counts of woodpeckers and bats were carried out in this habitat mosaic. It largely included managed forest (undisturbed by the windstorm), and also windthrow with varying levels of tree stand damage (from small gaps created by the storm to damage of the whole stand) and different level of salvage logging. The managed windthrow area was cleared from fallen and broken trees and artificially replanted afterwards. Consequently, young pine and birch plantations predominated here but also some small patches (<10 ha) of undamaged forest were still present at the site. The part of windthrow left without intervention was characterized by high abundance of dead standing, leaning or uprooted trees, fallen logs and occasional presence of undamaged forest patches. This habitat abounded in seedlings of pines, birches, oaks and spruces, especially in areas where canopy closure has been strongly reduced. There were no artificial nest-boxes set up for bats in the study area. To ensure that different forest habitats (both damaged and undisturbed as well as both salvaged and non-intervention) are representative in our sample, we distributed our point-count sites evenly in available habitats.

### Woodpecker sampling

The Great Spotted Woodpecker was inventoried in the spring of 2011. For this purpose, a total of 63 point-count sites were selected. The point-count sites were located regularly in a 300 m × 300 m grid, but some of them were slightly moved in order to exclude areas that were inaccessible (e.g., fenced against ungulates; Fig. 1). As a consequence, majority of the points were situated at least 200 m apart (mean distance to nearest point = 285 m, range: 186–453 m, SD = 45 m).

The counts of woodpeckers were conducted by one experienced observer using fixed-radius point count method (Gregory, Gibbons & Donald, 2004), which is one of the most commonly used technique to inventory and monitor bird populations and provide reliable index of bird abundance (Dunford & Freemark, 2005; Paquet et al., 2006; Venier & Pearce, 2007; Gregory et al., 2007; Greenberg et al., 2008; Żmihorski, Berg & Pärt, 2016). The order of sampling was random. Each point-count site was visited twice: in April and June during early mornings (from sunrise to 10 a.m. in April and to 9 a.m. in June). The counts were conducted only in good weather conditions (without heavy rain, strong wind or fog). Each visit lasted 10 min and during the visit all heard or seen individuals of the Great Spotted Woodpecker within a radius of approximately 100 m of the point-count site were counted. To ensure most reliable results, the fieldwork was preceded by a reconnaissance in the study area and training of distance estimation in the field, following the instructions given by Kepler & Scott (1981). To avoid double-counting of the same individuals (e.g., the same bird flying around) the observer took location of vocalizing individuals into account when interpreting the observations and assumed more than one individual only when it was obvious (e.g., birds vocalizing from different directions at the same time, visual observations of birds in different places, etc., Gregory, Gibbons & Donald, 2004). The individuals flying over tree tops were not recorded.

### Bat sampling

The study area was surveyed for bat activity in June and July 2011 from half an hour before dusk until half an hour before sunrise using frequency division bat detectors (AnaBat SD2, Titley Electronics) with the standard microphones. Recordings of bat activity were made at the same 63 point-count sites that were surveyed for woodpeckers (Fig. 1). No sampling was performed during strong wind or rainy conditions. Within the survey period, a total of seven nights of bat recordings were conducted at about weekly intervals, and during each night all of the 63 point-count sites were visited (i.e., each site was visited 7 times, 428 visits in total; in 13 cases it was not possible to do the inventories due to weather conditions). Each visit lasted for 10 min which is relatively short for bats, but enables coverage of many sites during short time period if not many detectors are available. During the visit, bat echolocation activity (i.e., normal echolocation during flight and rapid bursts of calls indicating foraging, so-called “feeding buzzes”) was recorded by the detector. The device was mounted on a tree, about 1 m above the ground (i.e., above the understory vegetation), with microphone pointed in the direction of bat’s expected flight path, that is, toward the area with the fewest number of obstructions, for example, trees. Each detector was set to a frequency-division ratio of 16 and a sensitivity level of 6–8. During each point visit we selected an optimal sensitivity value which allowed to maximize the quality of recordings and minimize insect interference and other noise. The data were recorded on a compact flash card via the AnaBat Zero-Crossing Analysis Interface Module. The sampling was performed by three operators and each of them visited the same point-count sites over the series of seven visits, each time starting from a different site.

### Bat call analysis

The acoustic data were analyzed using AnalookW software (Titley Electronics). The recordings were mostly sequences of a single individual ultrasonic echolocation calls, although there were also some records of simultaneous flight of more than one individual. The echolocation sequences were identified to species level by one researcher based on an examination of differences in the frequency of pulses emitted by bats. We used automatic filters available in the AnalookW software for identification of recordings containing bat echolocation calls. The calls were visualized by generating sonograms and classified based on characteristic frequency and other call parameters. Calls with a characteristic frequency in the range of 35–44 kHz were attributed to *P. nathusii*, 45–49 kHz to *P. pipistrellus* and 50–58 kHz to *P. pygmaeus* (Russ, 2012; modified by M. Zegarek to the range of characteristic frequency observed in northern Poland). Calls were identified as those of *Barbastella barbastellus* if they fitted the description given by Denzinger et al. (2001). Due to high similarity of the signals and possibility to misclassification, the records of other bat species were assigned to groups of several species. Two species, *Eptesicus serotinus* and *Vespertilio murinus*, emitting narrowband echolocation calls with characteristic frequency in the range of 24–33 kHz and pulse intervals of 150–200 ms, were considered as an *Eptesicus* and *Vespertilio* group. Calls of *Nyctalus noctula* and *Nyctalus leisleri* were included in a *Nyctalus* spp. group based on average intervals between pulses of more than 200 ms and characteristic frequency below 24 kHz (Vaughan, Jones & Harris, 1997). The last two groups: *Myotis* spp. (i.e., *Myotis daubentonii, Myotis nattereri*) and *Plecotus auritus* consisting of bat species that use short-duration (<2 ms) modulated signals were separated based on the characteristics given by Vaughan, Jones & Harris (1997) and Collins & Jones (2009). As an estimate of bat species richness we calculated the total number of species or species groups at each site per visit thus giving a maximum of five species/species groups. As an index of bat activity we calculated the total duration of all the call files recorded during a single night visit at a given point-count site for a given species/species group and for all the species pooled together. We used duration of echolocation as this measure correlates well with number of passes (*r* > 0.9), but provides more information: for a certain number of passes there is a variation in echolocation time, and this might be important for less common species.

### Statistical analysis

The associations between woodpecker abundance and indices of bat community were analyzed by using generalized additive mixed models (GAMM). In total 15 models were fitted and in all GAMMs a single bat sampling visit was a data record (i.e., 428 visits in total). First, we built a model (GAMM1) with Poisson error distribution explaining bat species richness per single 10-min visit. We used Poisson family as variance of number of species originated from counts (1.56) was nearly equal to the average (1.40). Second, we performed seven models with binomial error distribution explaining probability of bat presence (i.e., presence of echolocation calls during single 10-min visit; 1—present, 0—absent) for all species pooled (GAMM2) and separately for each species/group of species (GAMMs 3-8). Third, we performed seven models with gamma error distribution explaining duration of echolocation calls only for the subset of visits with bat present. We performed gamma models for all species pooled (GAMM9) and separately for each species/group of species (GAMMs 10-15). Gamma family was chosen as duration of echolocation is continuous, always positive and right-skew.

We considered three explanatory variables in each model: (1) Julian date (ranging from 162 to 207; continuous variable), (2) time of a day (ranging from 19:00 p.m. to 3:30 a.m.; continuous variable fitted with spline) and (3) abundance of the Great Spotted Woodpecker; that is, the total number of woodpecker individuals recorded in two survey visits performed at a given point-count site. Including Julian date and time of day was necessary to account for temporal patterns in bat activity and to make observed bat diversity comparable among visits. In case of time of day we used thin plate regression spline allowing for non-linear fit estimated directly from data but linear fit is also possible if data do not support non-linear relationship (Wood, 2017). We used spline in this case as temporal activity in bats is variable and often shows non-linear pattern (Ruczyński et al., 2017). In the spline fit we set k value (indicating allowed wiggliness of the fit; *k* = 1 indicates straight line) to 5 and used “*k*.check” function for diagnostic of the basis dimension selected and the test confirmed that *k* values in all significant spline fits were adequate. As at each point-count site seven visits were performed, we introduced site identifier as a random factor in all 15 GAMMs to account parameter estimates for possible dependency of observations taken in the same point-count sites. We present parameter estimations from the full models fitted in “mgcv” package (Wood, 2017) in R (R Core Team, 2018).

All 15 models were checked for the residual spatial autocorrelation using spline correlogram plotted using “ncf” package (Bjornstad, 2018) in R, and showing correlation among residuals as a nonlinear function of spatial distance between these data records. In no case the correlogram indicated spatial dependance of the residuals, thus confirming spatial dependency of the data is not a problem in our study. Also, we estimated goodness of fit of the performed models by calculating residual deviance to residual degrees of freedom ratio. Following Bilder & Loughin (2015) we assumed that the ratio may indicate a poor fit when exceeds 1 + 3(2/residual d.f.)^0.5^.

## Results

During all the surveys performed 54 individuals of the Great Spotted Woodpecker were recorded within a 100 m radius of the 63 point-count sites sampled. The total number of woodpecker individuals observed at a given site in two survey visits ranged from 0 to 5 (mean ± SE = 0.86 ± 0.14).

**Figure 2:**
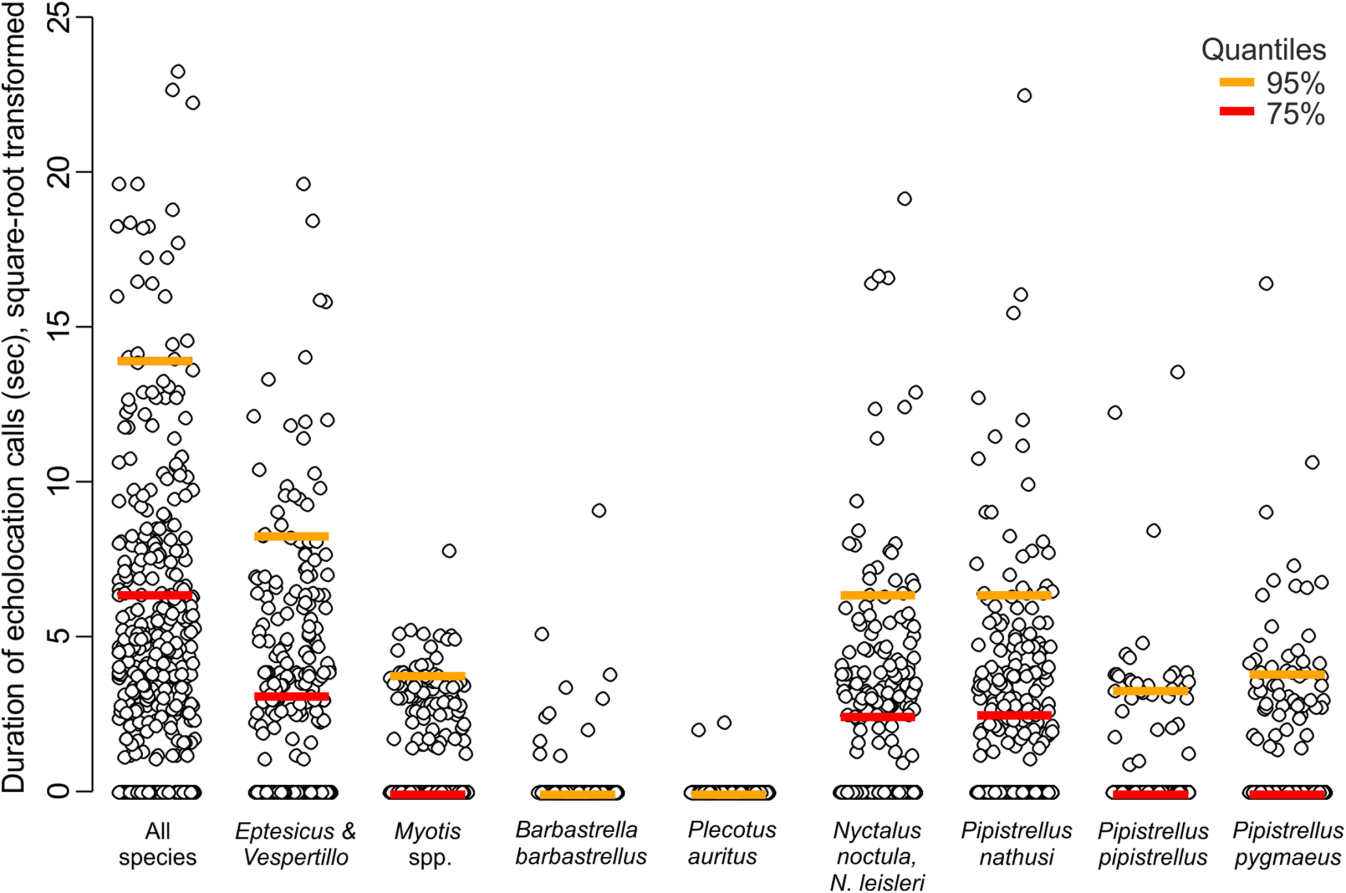
Duration of echolocation calls of eight bat species or species groups recorded at 63 sites in pine forest in NE Poland during 428 visits performed in 2011.

**Figure 3:**
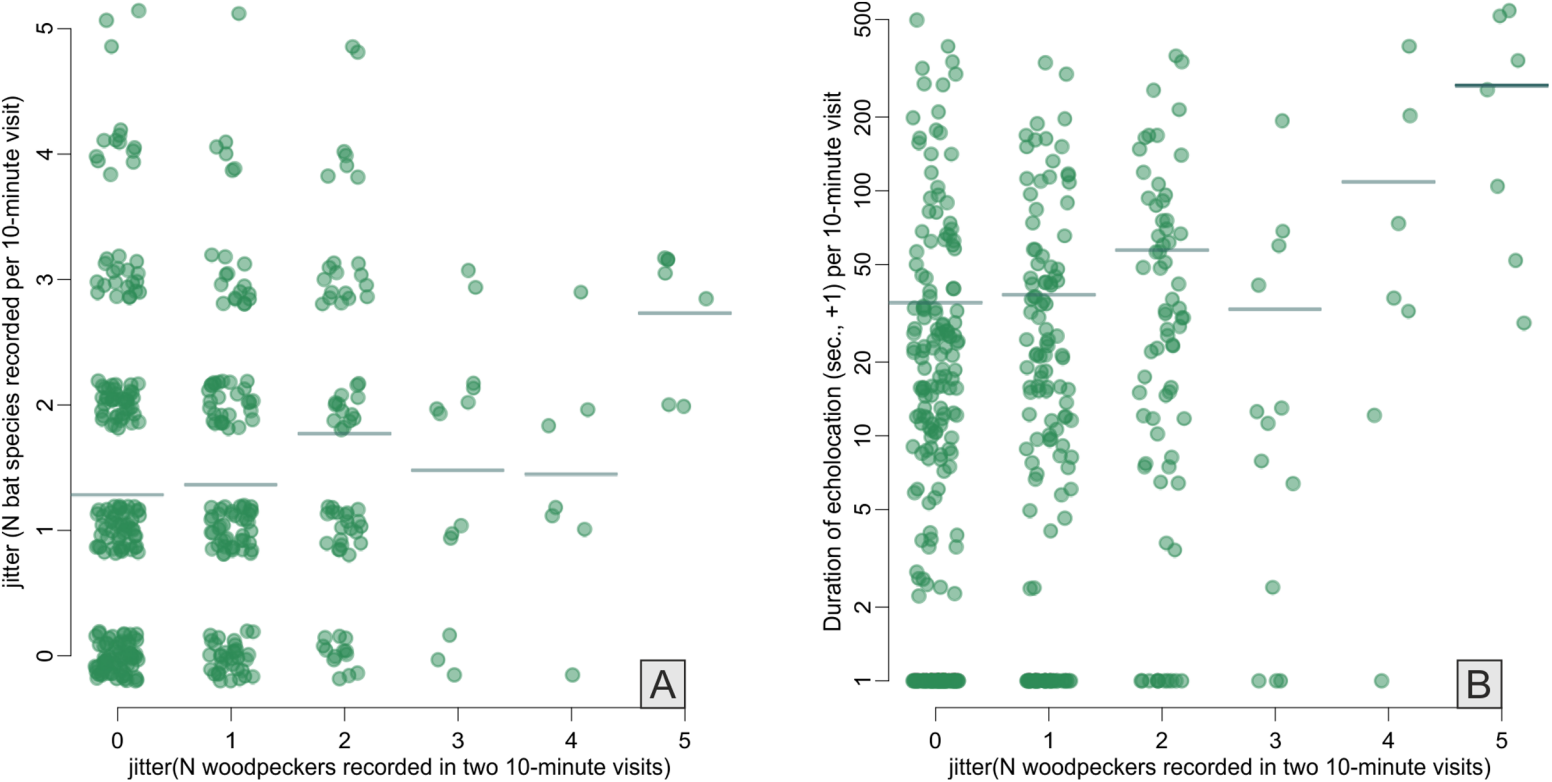
Correlations between Great Spotted Woodpecker abundance (*x*-axes) and number of bat species recorded (A) and duration of bat echolocation (B). Random noise was added to points to avoid symbols overplotting, horizontal lines indicate means. Note log+1 *y*-axis transformation in B.

Across the bat survey period, we recorded a total of 18,135 s of bat echolocation calls of eight species/species groups including: brown long-eared bat *Plecotus auritus*, barbastelle bat *Barbastella barbastellus*, Nathusius’ pipistrelle *Pipistrellus nathusi*, common pipistrelle *Pipistrellus pipistrellus*, soprano pipistrelle *Pipistrellus pygmaeus*, unidentified *Nyctalus* spp., *Eptesicus* and *Vespertilio* group and unidentified *Myotis* spp. Hereafter we refer to these groups as species. There were also some records of bat calls that were not possible to assign to any species or group of species (see Appendix S2). Bats occurred in about 71% of visits. Among recorded species the *Eptesicus* and *Vespertilio* group and *Pipistrellus nathusi* were the most frequently observed (Fig. 2; Appendix S2). The pooled duration of bat calls detected during a 10-min visit ranged between 0 and 542 s with a mean of 42.37 ± 3.78 s and the number of bat species recorded during a visit ranged between 0 and 5 (mean = 1.40 ± 0.06). Both the bat species richness and duration of bat echolocation were positively correlated with the local Great Spotted Woodpecker abundance (Pearson’s correlation, *r* = 0.16, *t* = 3.33, df = 426, *p* < 0.0001; *r* = 0.28, *t* = 5.96, df = 426, *p* < 0.0001, respectively, Fig. 3).

**Figure 4:**
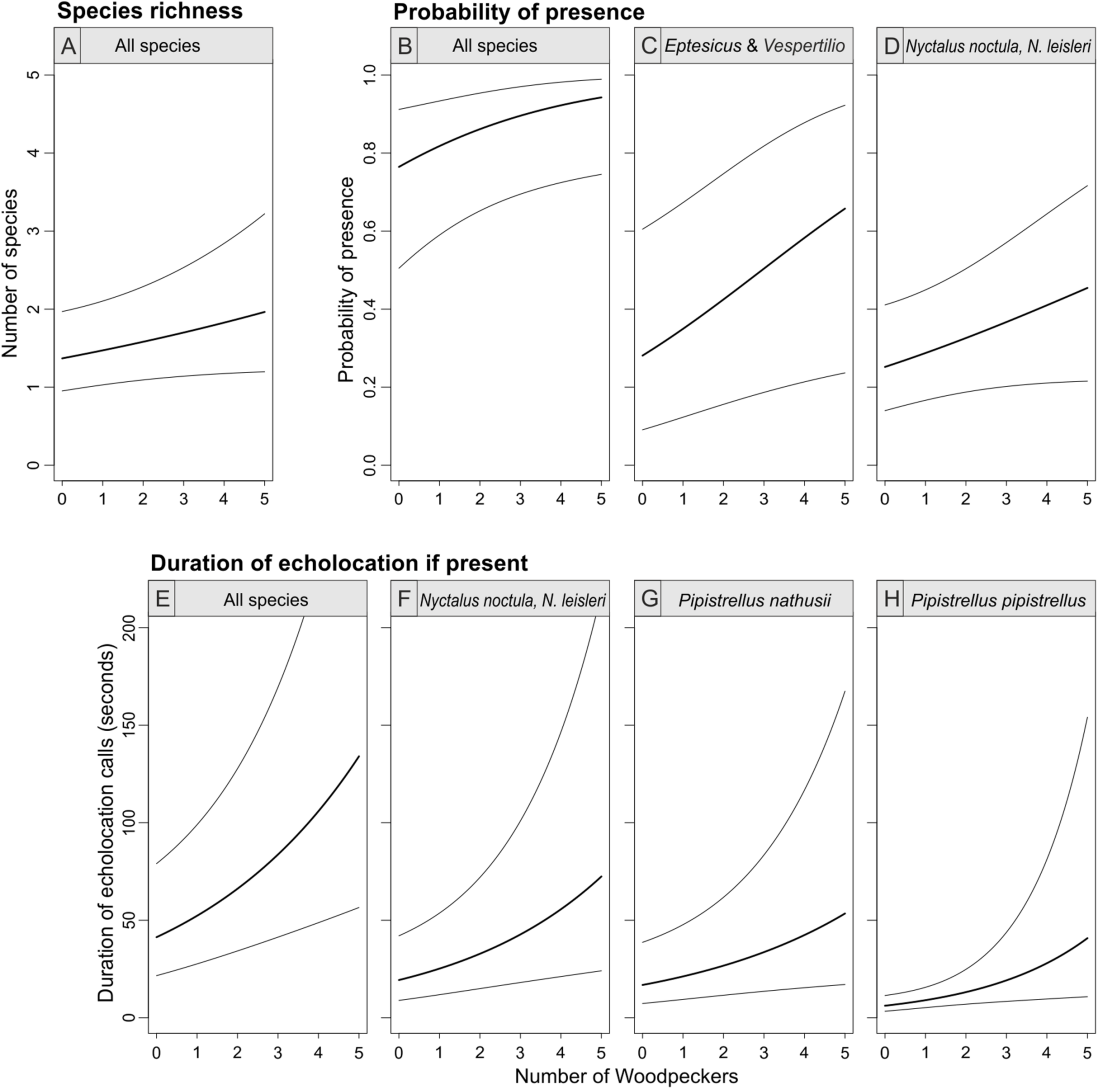
Bat species richness (A), probability of presence (B–D) and duration of echolocation calls (E–H) as a function of number of Great Spotted Woodpeckers observed at a point-count site in Pisz Forest, NE Poland, as predicted by models summarized in Table 1. Thick curves represent mean, thin curves show 95% confidence intervals. Predictions are made for hour = 23:00 and day of year = 200.

The abundance of the Great Spotted Woodpecker was a positive predictor of bat species richness (with 95% confidence interval of the parameter slightly overlapping zero), probability of bat presence and pooled activity of all bat species (duration of all echolocation calls; Table 1; Fig. 4). The occurrence of *Eptesicus* and *Vespertilio* bats and *Nyctalus* species was positively related with the number of woodpecker individuals. The activity of *Eptesicus* and *Vespertilio* group and *Myotis* species, was not correlated with the abundance of the woodpecker. In contrary, *Nyctalus* bats were positively associated with woodpeckers, also *Pipistrellus* species showed positive correlation (with except of *P. pygmaeus*, Table 1; Fig. 4).

**Table 1:** Summary of generalized additive mixed models explaining bat species richness, probability of bat presence and duration of echolocation in the pine forest, NE Poland in relation to abundance of the Great Spotted Woodpecker, day of year and time of day. Parameter estimates accompanied by 95% CIs in brackets are given for linear fit and estimated degrees of freedom (edf) in case of spline fit. Models’ goodness of fit is given in last column (GoF) as a ratio between residual deviance and residual degrees of freedom. Significant and marginally insignificant (*p* < 0.1) eﬀects are marked in bold. The significance levels (*p*-values) are explained at the bottom.

Model no.	Response variable	Explanatory variables			Deviance explained (%)	GoF
		Woodpecker	Day of year	Time of day		
Species richness (Poisson GAMM)						
GAMM1	All species	**0.072 (−0.010 to 0.154)^^^**	0.010 (0.004 to 0.015)***	edf = 3.15***	20.0	1.11
Probability of presence (Binomial GAMM)						
GAMM2	All species	0.324 (0.019–0.628)*	0.032 (0.015–0.048)***	edf = 3.60***	20.6	0.94
GAMM3	*Eptesicus, Vespertilio*	0.318 (0.021–0.615)*	0.054 (0.037–0.070)***	edf = 2.93***	29.7	0.95
GAMM4	*Myotis* spp.	−0.029 (−0.339 to 0.280)	−0.013 (−0.029 to 0.003)	edf = 2.53	17.3	0.90
GAMM5	*Nyctalus noctula, N. leisleri*	**0.181 (−0.027 to 0.390)^^^**	−0.004 (−0.018 to 0.010)	edf = 1.00***	7.1	1.11
GAMM6	*Pipistrellus nathusii*	0.181 (−0.053 to 0.415)	0.009 (−0.005 to 0.022)	edf = 1.00***	12.4	1.11
GAMM7	*Pipistrellus pipistrellus*	0.221 (−0.055 to 0.497)	0.028 (0.004–0.050)*	edf = 1.00**	8.3	0.54
GAMM8	*Pipistrellus pygmaeus*	−0.491 (−1.512 to 0.530)	0.043 (0.017–0.067)***	edf = 1.08***	50.8	0.41
Duration of echolocation if present (Gamma GAMM)						
GAMM9	All species	0.235 (0.095–0.375)**	0.018 (0.010–0.026)***	edf = 2.59***	39.1	1.07
GAMM10	*Eptesicus, Vespertilio*	−0.090 (−0.259 to 0.079)	**0.011 (−0.001 to 0.024)^^^**	edf = 1.16***	28.5	0.99
GAMM11	*Myotis* spp.	0.009 (−0.122 to 0.141)	0.001 (−0.007 to 0.010)	**edf = 1.16^^^**	20.6	0.40
GAMM12	*Nyctalus noctula, N. leisleri*	0.264 (0.068–0.459)**	0.025 (0.010–0.038)***	edf = 1.00**	51.8	0.80
GAMM13	*Pipistrellus nathusii*	0.231 (0.033–0.428)*	0.009 (−0.002 to 0.020)	edf = 1.00**	53.9	0.84
GAMM14	*Pipistrellus pipistrellus*	0.377 (0.080–0.673)*	0.015 (0.003–0.027)*	edf = 1.00	99.0	0.07
GAMM15	*Pipistrellus pygmaeus*	−0.142 (−0.509 to 0.225)	0.002 (−0.013 to 0.016)	**edf = 1.55^^^**	56.5	0.68

**Notes:**

^ 0.05 < *p*-value < 0.1.

* 0.01 < *p*-value < 0.05.

** 0.001 < *p*-value < 0.01.

*** *p*-value < 0.001.

**Figure 5:**
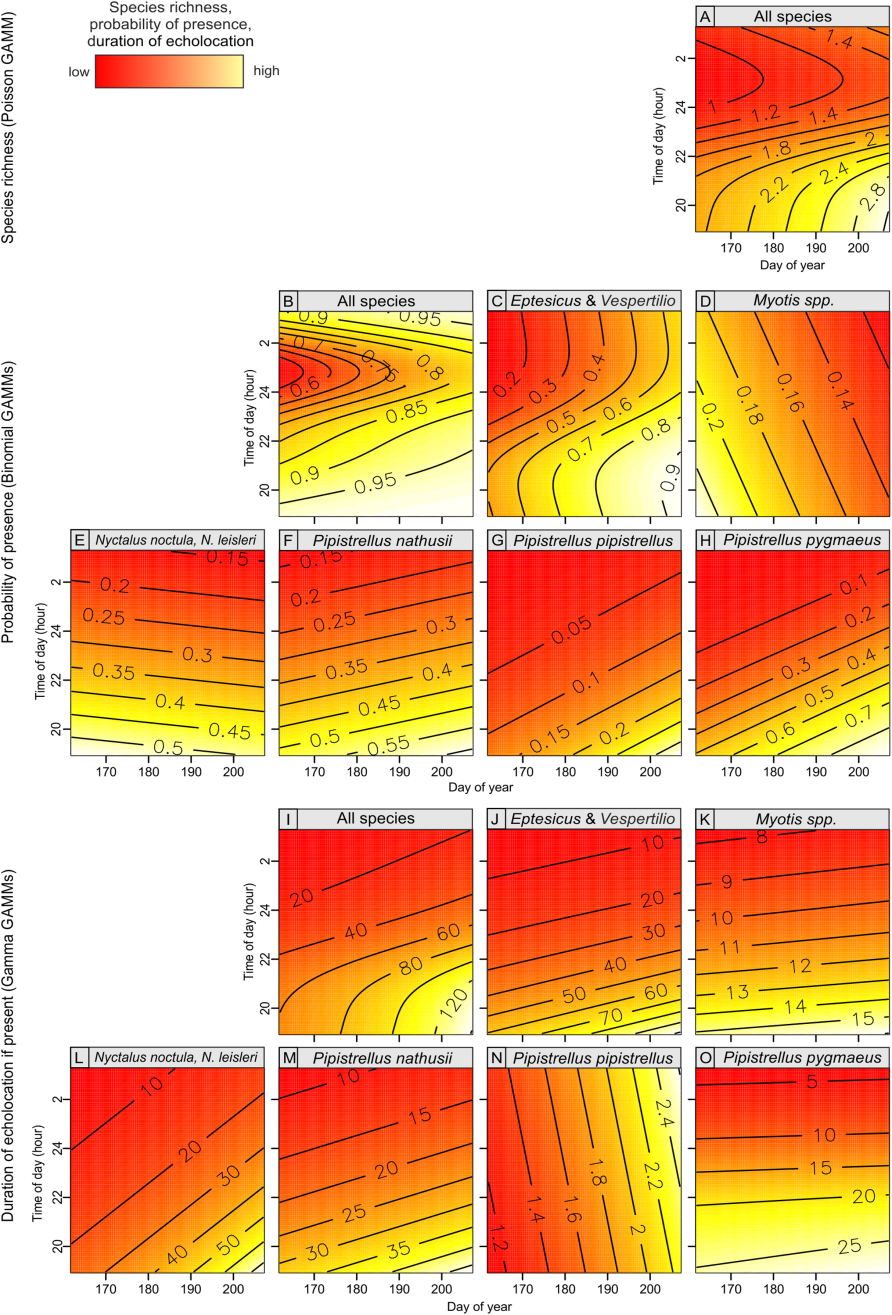
Bat species richness (A), probability of presence (B–H) and duration of echolocation calls (I–O) in relation to day of year and time of day, as predicted by models summarized in Table 1.

Bat species richness, occurrence and duration of echolocation of bats in most cases were significantly associated with day of year and time of day and were generally lower at the beginning of the summer season (i.e., mid June) but were increasing over time till end of July (Table 1; Fig. 5). The effect of time of day was also significant and in most cases the activity of single species was linearly decreasing from dusk (hour 19:00) to dawn (Table 1; Fig. 5). However, in case of pooled activity, occurrence and overall species richness the association was non-linear: it was decreasing from dusk but after ca. 1–2 am started to increase again (Table 1; Fig. 5). In two cases (models GAMM5 and GAMM6) relatively higher residual deviance to residual d.f. ratio indicating models’ goodness of fit may indicate potential problems but in all cases the ratio was below the threshold indicating a poor fit.

## Discussion

Our study is the first to investigate whether woodpeckers may be used as indicator species for forest living bat communities and we showed a positive correlation between the abundance of the Great Spotted Woodpecker and bat community richness, as well as occurrence and acoustic activity of several bat species. Thus, the Great Spotted Woodpecker seems to be a positive indicator of not only forest birds (De Gasperis et al., 2016) but also of bats. However, not all bat species or groups of species show the same correlation with the woodpecker. Below we discuss possible drivers of the observed patterns.

The positive association between woodpeckers and bats (both occurrence and acoustic activity) observed in our study could be possibly due to relatively high variation in habitat structure. Our survey covered sites differing in terms of amount of dead trees, which are important substrate for both woodpeckers and bats (in naturally regenerating windthrow or in old growth stands the amount of dead wood is usually much higher than in young plantations). Natural disturbances occurring in forest ecosystems, like windstorms or fires, have been reported to increase habitat heterogeneity and amount of dead wood (Drever et al., 2006; Gustafsson et al., 2019). Also in our study area the windstorm locally created numerous tree microhabitats including loose bark, cracks and natural holes, as well as high amount of dead and decaying wood in the form of standing snags, fallen logs and broken trunks. Such structural complexity may be important for a variety of forest specialists like woodpeckers, but also for bats as roosting habitats. At other sites, however, such roosts may have been missing. Such an explanation is in line with results described by Regnery et al. (2013) who showed that the diversity or density of tree microhabitats in the Mediterranean forest ecosystems were positively linked with both bird and bat community indices. The created openings in forest canopy could additionally increase prey accessibility for bat species that are unable to hunt in dense vegetation (e.g., *Nyctalus* spp., Müller et al., 2012) and consequently lead to increased foraging activity (Kusch et al., 2004). In present study, however, we focused at spatial association between woodpeckers and bats without taking forest characteristics into account, mainly due to logistic limitations. Identifying habitat elements and structures driving the spatial overlap of woodpeckers’ and bats’ abundances should be another step important for the understanding why bats and woodpecker co-occur and whether the co-occurrence depends on certain ecosystem characteristics.

Despite bats occupying both natural nest holes and artificial roosts in nest boxes (Berthinussen, Richardson & Altringham, 2014), to date very few studies have reported links between presence of bats and cavity-nesting birds. Myczko et al. (2017) have reported the spring reproductive coexistence of Starling *Sturnus vulgaris* and Noctule Bats *Nyctalus noctula* in natural nest-holes, but the co-occurrence of bats and woodpeckers remains understudied. Woodpeckers’ excavating activity, providing habitats crucial for species that require holes for nesting but are not able to create their own cavities (i.e., secondary-cavity nesters), has been broadly discussed so far (Drever et al., 2008; Martin, Aitken & Wiebe, 2004). Thus, the spatial association between bats and the Great Spotted Woodpecker could be also due to the fact that the cavities excavated by the woodpecker may be utilized by roosting bats (Ruczyński & Bogdanowicz, 2005). Furthermore, woodpeckers eat mainly larvae of insects developing in dead wood (e.g., beetles) during the breeding season (Cramp, 1985) which in the second part of the season may be an important part of the bats’ diet (as was also discussed by Tillon et al. (2016)). Thus, locally elevated invertebrate food resources, related to highly heterogeneous habitats, like windthrows (Wermelinger et al., 2017), can probably attract both insectivorous groups (Barbaro et al., 2019), resulting in positive spatial correlation among woodpeckers and bats. On the other hand, the Great Spotted Woodpecker is considered an efficient robber of birds’ nests located in cavities, thus may contribute to brood loss (Czeszczewik & Walankiewicz, 2003; Walankiewicz, 2002) and similar impact on bats cannot be excluded (Sparks et al., 2003). As a consequence, this negative interaction may relax spatial co-occurrence between woodpeckers and bats.

In our study area *Eptesicus* and *Vespertilio* group was most likely dominated by Serotine bat *Eptesicus serotinus* which is a synanthropic species that does not breed in forests, but often forages along forest roads. Thus, sites rich in tree cavities (both natural ones and those excavated by the woodpecker) do not necessarily result in higher activity of these bat species, although we recorded positive correlation between the woodpecker and presence of these bat species. *Myotis* spp. group comprises forest species that breed in forest and tree hollows and one may thus expect activity of these species to be correlated with woodpecker abundance. We did not, however, confirm such relationship (both for occurrence and echolocation duration), which can be caused by strictly technical issues—bat detectors we used have rather low detectability in case of species belonging to this group. In contrary, *Nyctalus* bats were associated with the woodpecker abundance, which confirms our expectations as these species are typical of forests and use tree cavities for roosting and breeding. Also the smallest ones, *Pipistrellus* bats, use tree hollows for breeding and thus can benefit from woodpeckers excavating activity. Interestingly, occurrence of *Pipistrellus* species was not associated with the woodpecker abundance but at sites occupied by these bats duration of their echolocation was positively linked with abundance of the woodpecker.

Finally, we also recorded clear temporal variation of studied bat community. Generally, bats were most often present and acoustically active just after sunset, and activity was decreasing toward midnight. This result generally confirms earlier findings concerning temporal variation of bat activity, which can be driven by both insect biomass and air temperature (Hayes, 1997; Ruczyński et al., 2017). Also, more bats were recorded later in the season which may be associated with the appearance of newly weaned juveniles (Ciechanowski et al., 2010). Furthermore, recent studies showed that activity of nocturnal insects in different habitats of temperate Europe is increasing during June and peaks around mid-July (Ruczyński et al., 2020) which also can drive seasonal bat activity (Hayes, 1997).

## Conclusion

The abundance of the Great Spotted Woodpecker may indicate a rich habitat for bats. Although mechanism causing this association needs further studies taking habitat variables into account, we suspect that presence of woodpeckers can be linked with higher availability of breeding or roosting sites (i.e., cavities created by woodpeckers and natural tree holes) and food resources (saproxylic invertebrates) for bats. Nevertheless, this suggests that the Great Spotted Woodpecker may function as an indicator of forests with high activity of bat species which can be useful for inventories of these mammals in pine-dominated forests. Such positive correlation could also have important consequences for management (woodpeckers are diurnal species and thus are easier to detect than cryptic bats) and conservation (protection of woodpecker can benefit bats) and we, therefore, recommend taking this association into account when planning, for example, bat monitoring.

## Supplemental Information

10.7717/peerj.9385/supp-1 Supplemental Information 1 Different habitats of Pisz Forest. (A) Undisturbed managed forest. (B) Salvaged windthrow. (C) Naturally regenerating windthrow.

10.7717/peerj.9385/supp-2 Supplemental Information 2 Range, mean values and SE of bat species activity recorded during a 10-min visit and proportion of visits in which the bat species were observed. The index of bat activity was calculated as the duration of echolocation calls in seconds. The visits were performed at 63 point-count sites in 2011 in the Pisz Forest.

10.7717/peerj.9385/supp-3 Supplemental Information 3 Raw data.

## Additional Information and Declarations

### Data Availability

The following information was supplied regarding data availability: The raw data are available in a Supplemental File.
