# Supplementary figures and images for: Spatial patterns of bat diversity overlap with woodpecker abundance
Source: PeerJ. 2020 Jun 18;8:e9385. doi: 10.7717/peerj.9385 (PMC7306217; doi:10.7717/peerj.9385)

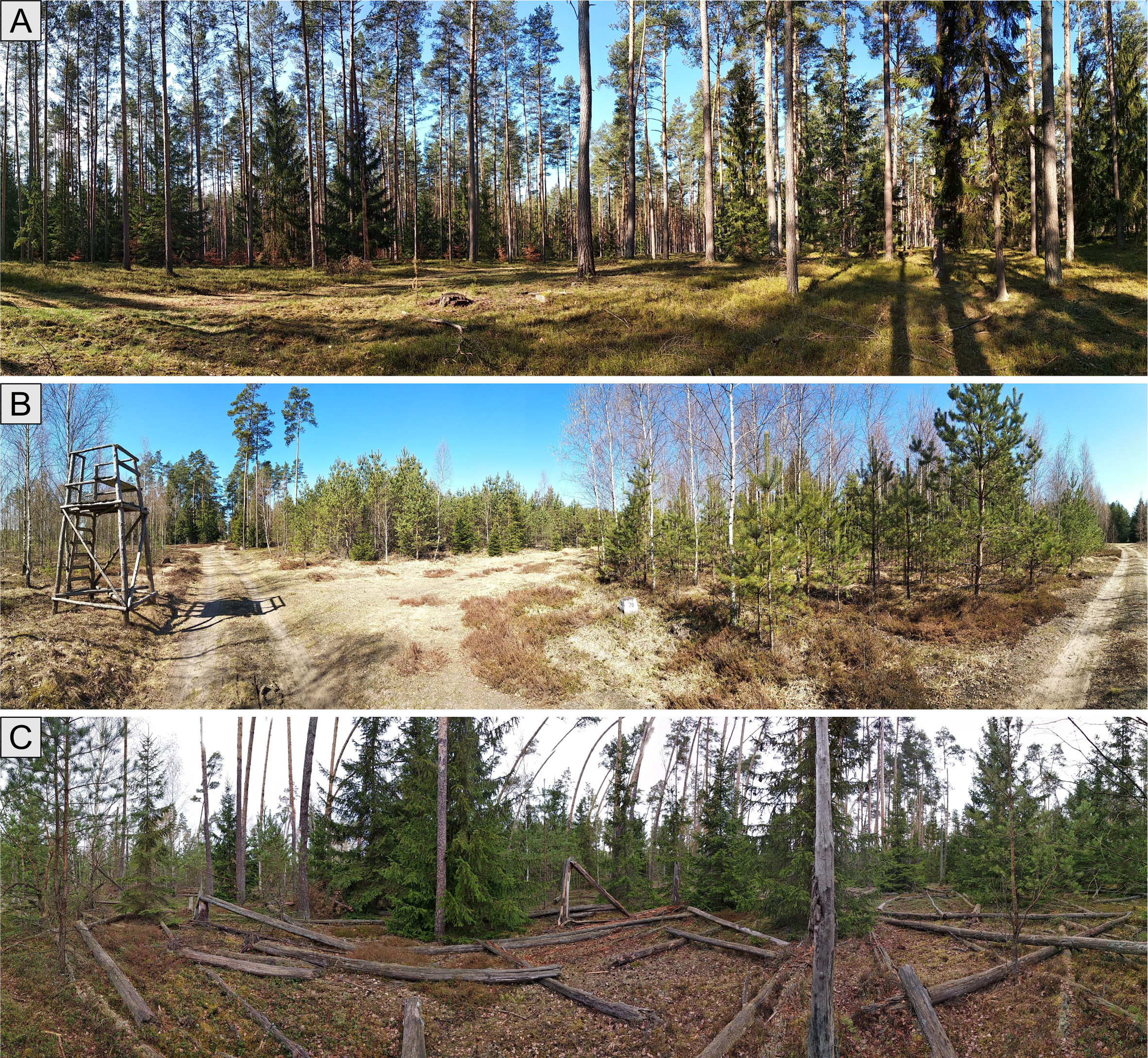

Supplement: Supplemental Information 1 — (A) Undisturbed managed forest. (B) Salvaged windthrow. (C) Naturally regenerating windthrow. [file peerj-08-9385-s001.png]
