# Supplementary material for: Spatial patterns of bat diversity overlap with woodpecker abundance
Source: PeerJ. 2020 Jun 18;8:e9385. doi: 10.7717/peerj.9385 (PMC7306217; doi:10.7717/peerj.9385)
Supplement: Supplemental Information 2 — The index of bat activity was calculated as the duration of echolocation calls in seconds. The visits were performed at 63 point-count sites in 2011 in the Pisz Forest. [file peerj-08-9385-s002.docx]

| Species/Species group | Duration of echolocation | | Frequency of occurrence (proportion of visits) |
| --- | --- | --- | --- |
|  | Range | Mean ± SE |  |
| *Eptesicus,*  *Vespertilio*^a^ | 0-386.25 | 14.23 ± 1.92 | 0.33 |
| *Pipistrellus nathusi* | 0-506.89 | 9.68 ± 1.69 | 0.33 |
| *Nyctalus*^b^ | 0-367.48 | 9.40 ± 1.64 | 0.29 |
| *Pipistrellus pygmaeus* | 0-270.31 | 2.97 ± 0.77 | 0.13 |
| *Myotis*^c^ | 0-61.10 | 2.35 ± 0.29 | 0.21 |
| *Pipistrellus pipistrellus* | 0-184.51 | 1.80 ± 0.60 | 0.09 |
| *Barbastella barbastellus* | 0-83.11 | 0.39±0.21 | 0.03 |
| *Plecotus auritus* | 0-5.24 | 0.02±0.01 | 0.00 |
| Unidentified | 0-100.65 | 1.52 ± 0.34 | 0.12 |

^a^ *Eptesicus serotinus* and *Vespertilio murinus*

^b^ *Nyctalus noctula* and *N. leisleri*

^c^ *Myotis daubentonii* and *M. nattereri*
